# Correction: A biomimetic approach to shielding from ionizing radiation: The case of melanized fungi

**DOI:** 10.1371/journal.pone.0257068

**Published:** 2021-08-31

**Authors:** Thomas Vasileiou, Leopold Summerer

Fig 2 is incorrect. Specifically, Fig 2A, which depicts the different geometric configurations discussed in the article, plots the axis defining the parameter *h*_*r*_ in reverse and shows the examples of the two extreme “film positions” (marked as *h*_*r*_ = 0 and *h*_*r*_ = 1 in panel (A)) in the opposite order. The authors have provided a corrected version here.

As a result, in the third paragraph of the subsection “Spatial arrangement of melanin affects shielding effectiveness” in the “Results”, the phrase reading “We repeated the simulations for the aforementioned values of *ρ*_*A*_ for two configurations: melanin ghosts and film with *h*_*r*_ = 0.” should be corrected to “We repeated the simulations for the aforementioned values of *ρ*_*A*_ for two configurations: melanin ghosts and film with *h*_*r*_ = 1.”.

**Fig 2:**
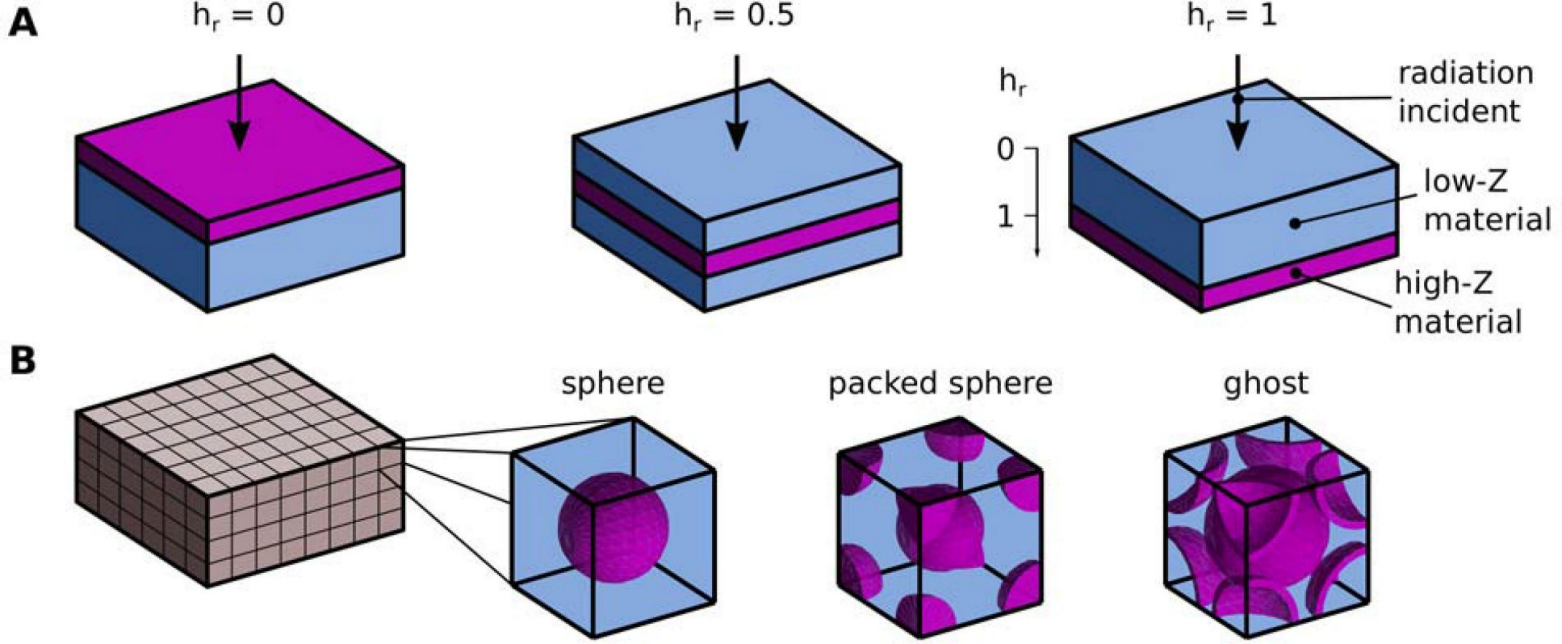
Schematic of the simulated spatial arrangements. (A) Illustrations of the film spatial arrangement, at three relative positions (*h*_*r*_ = 0, 0.5 and 1). The direction of the incoming radiation is indicated by the arrow. (B) Illustration of the lattice spatial arrangement for three configurations: sphere, packed sphere and ghost.
