# Supplementary material for: A biomimetic approach to shielding from ionizing radiation: The case of melanized fungi
Source: PLoS One. 2020 Apr 24;15(4):e0229921. doi: 10.1371/journal.pone.0229921 (PMC7182175; doi:10.1371/journal.pone.0229921)
Supplement: S1 Text — (PDF) [file pone.0229921.s001.pdf]

Supporting information for "A biomimetic approach to shielding from ionizing radiation: the case of melanized fungi"

Thomas Vasileiou*, Leopold Summerer

ESA - Advanced Concepts Team, European Space Research and Technology Centre (ESTEC), NL-2200AG Noordwijk, Netherlands

1 Selection of radioactive source

Previous experiments [1–3], which have proposed the interaction between melanin and Compton electrons, employed the ^{137}Cs radioactive source, a γ-emitter with energy of $E_\gamma = 0.662\,\text{MeV}$. The ^{60}Co source—a γ-source with its main decay path emitting two photon with $E_\gamma = 1.173\,\text{MeV}$ and $E_\gamma = 1.332\,\text{MeV}$—has also been employed to test the effect of radiation in animal models [4, 5]. In our experiments, we seek to reproduce the energy spectrum of the secondary Compton electrons from these sources. The energy of the recoil electron, E_e, depends on the scattering angle θ as [6]

$$E_e = E_\gamma \left[1 - \frac{1}{1 + \frac{E_\gamma}{m_e c^2}(1 + \cos\theta)} \right] \tag{1}$$

where m_e is the electron resting mass and c is the speed of light. Hence, the maximum value for E_e is given for back scattering of the incident photon ($\theta = \pi$), which in the case of ^{137}Cs and ^{60}Co are equal to $0.477\,\text{MeV}$ and $1.118\,\text{MeV}$ respectively. Assuming that the differential cross section of the scattered photons follows the Klein–Nishina distribution, the spectrum of E_e can be estimated analytically. The lengthy derivation is provided in [6]. This analytic treatment applies for mono-energetic photon scattering; in reality the outgoing photon may repeat the inelastic scattering process multiple times, skewing the spectrum towards the low energy band. Still, the analytic approximation gives a good basis for selecting the β-source.

S1 Fig compares the theoretical energy spectrum of the Compton electrons for the ^{137}Cs and ^{60}Co sources. We note that for the ^{137}Cs source we assumed that all photons have same E_γ, whereas for the ^{60}Co source we assumed that photons have a 50 % chance being at each of the two E_γ. We also plot the spectrum of ^{90}Sr source [7], which exhibits significant energy content in the area of interest.

2 Simulation details

This section give a detailed description on the parameters, the materials and the geometries used in Geant4 simulations. For the secondary particle production, we set the range cut at $2\,\mu\text{m}$ (which defines a material depended energy threshold for secondary particle production) and the universal low and high energy limits at $250\,\text{eV}$ and $100\,\text{GeV}$ respectively. We activated the atomic de-excitation physics for fluorescent and Auger electron and we deactivated the particle induced X-ray emission. For comparison to the shielding experiments, the detector was simulated as a $10\,\text{mm} \times 10\,\text{mm} \times 5\,\text{mm}$ $Cd_{0.9}Zn_{0.1}Te$ crystal covered with a $22\,\text{mm} \times 22\,\text{mm} \times 0.5\,\text{mm}$ aluminum cover placed at $3.65\,\text{mm}$ from the top of the crystal (values from manufacturer).

The composition of the shielding materials are given in Table S1. For the synthetic melanin the suppliers provides the weight fraction for carbon and nitrogen. The rest of the values were

adjusted from [8], where the authors provide the elemental analysis of melanin from the same supplier. The melanin from *S. officinalis* has a more complex composition containing both metals and amino-acids [9] and has been analyzed by various studies. The values reported in Table S1 are the mean values for the weight fraction from [8, 10, 11], given that the values for the each element was provided in the respectful study. The density for melanin was set to $1680\,\mathrm{kg\,m^{-3}}$ [12].

The chemical formula for the cellulose nano-crystal has been determined by the supplier to be $(C_6H_{10}O_5)_x(C_6H_9O_4SO_4Na)_y$. Elemental analysis performed by a different group on samples from the same supplier determined the sulfur content to be 1.1 wt. % [13]. These two criteria determine the elements and the respective weight fraction for the cellulose nano-crystals.

3 Simulated vs. experimental spectra

For the shielding experiments, the simulated spectra do not match the ones recorded during the experiments, with the latter having more content in the low energy region. To investigate further, we performed the same comparison in the absence of a sample, namely with the radioactive source and the detector only present. Panel A of S4 Fig contrasts the simulates spectrum, labeled as “Devaney”, to the experimental, labeled as “exp”. Since the same trend can be observed also in this case, we conclude that the energy shift is not connect to the presence of melanin, the solvent or the container.

We speculate that the observed discrepancy is due to the modeling of the detector and specifically the way the deposited energy is registered. In our simulations, E_d contain the contribution of the deposited energy from the primary and all the secondaries particles. This simplifying assumption inaccurately captures the exact detector dynamics, which are determined by the electronics and the signal processing algorithms that are applied to the voltage pulses induced in the crystal by the incoming particles. To further investigate if the neglected dynamics are able to produce the magnitude of the observed discrepancy on the energy spectra, we repeated the simulations with a detector model that records E_d for every particle separately, without taking into account if the particle is primary or secondary. We employed two ways to simulate the ^{90}Sr source: a) as an electron emitter with spectrum taken from [7], as previously, and b) as a collection of immobile ^{90}Sr nuclei with the radioactive decay module of Geant4. The latter tracks all the intermediate nuclear reactions and products, making it computationally more demanding. A comparison of the two detector models is presented in S4 Fig. Both simulate spectra, from [7] and the radioactive decay module Geant4, produce similar results. More importantly, the neglected detector dynamics are able to produce the observed shift in the energies between simulation and experiment, with the real detector response most likely lying somewhere between these two extreme cases.

The selected detector model captures the essence of the shielding experiments while keeping the modeling complexity and the execution time acceptable, in the expense of accuracy of the final spectrum. The calculation of D is not affected by the detector modelling, since in the simulations the production and tracking of the secondary particles is not affected by the detector dynamics. We only expect a minor effect on the estimation of σ_D^2.

References

1. Dadachova E, Bryan RA, Huang X, Moadel T, Schweitzer AD, Aisen P, et al. Ionizing radiation changes the electronic properties of melanin and enhances the growth of melanized fungi. PloS one. 2007;2:e457. doi:10.1371/journal.pone.0000457.

2. Schweitzer AD, Howell RC, Jiang Z, Bryan RA, Gerfen G, Chen CC, et al. Physico-chemical evaluation of rationally designed melanins as novel nature-inspired radioprotectors. PloS one. 2009;4(9):e7229.

3. Revskaya E, Chu P, Howell RC, Schweitzer AD, Bryan RA, Harris M, et al. Compton scattering by internal shields based on melanin-containing mushrooms provides protection of gastrointestinal tract from ionizing radiation. Cancer Biotherapy and Radiopharmaceuticals. 2012;27(9):570–576.

4. Kunwar A, Adhikary B, Jayakumar S, Barik A, Chattopadhyay S, Raghukumar S, et al. Melanin, a promising radioprotector: Mechanisms of actions in a mice model. Toxicology and applied pharmacology. 2012;264(2):202–211.

5. Rageh MM, El-Gebaly RH, Abou-Shady H, Amin DG. Melanin nanoparticles (MNPs) provide protection against whole-body γ-irradiation in mice via restoration of hematopoietic tissues. Molecular and cellular biochemistry. 2015;399(1-2):59–69.

6. Blumenthal GR, Gould RJ. Bremsstrahlung, synchrotron radiation, and Compton scattering of high-energy electrons traversing dilute gases. Reviews of Modern Physics. 1970;42(2):237.

7. Devaney JJ. Beta spectra of ^{90}Sr and ^{90}Y. Los Alamos National Lab; 1985.

8. Chedekel M, Murr B, Zeise L. Melanin standard method: empirical formula. Pigment cell research. 1992;5(3):143–147.

9. Liu Y, Hong L, Kempf VR, Wakamatsu K, Ito S, Simon JD. Ion-exchange and adsorption of Fe(III) by sepia melanin. Pigment cell research. 2004;17(3):262–269.

10. Mbonyiryivuze A, Nuru Z, Ngom BD, Mwakikunga B, Dhlamini SM, Park E, et al. Morphological and chemical composition characterization of commercial sepia melanin. American Journal of Nanomaterials. 2015;3(1):22–27.

11. Liu Y, Hong L, Wakamatsu K, Ito S, Adhyaru B, Cheng CY, et al. Comparison of structural and chemical properties of black and red human hair melanosomes. Photochemistry and Photobiology. 2005;81(1):135–144. doi:10.1562/2004-08-03-RA-259.1.

12. Ninh C, Cramer M, Bettinger CJ. Photoresponsive hydrogel networks using melanin nanoparticle photothermal sensitizers. Biomaterials science. 2014;2(5):766–774.

13. Nagalakshmaiah M, El Kissi N, Dufresne A. Ionic compatibilization of cellulose nanocrystals with quaternary ammonium salt and their melt extrusion with polypropylene. ACS applied materials & interfaces. 2016;8(13):8755–8764.
