# Supplementary figures and images for: A biomimetic approach to shielding from ionizing radiation: The case of melanized fungi
Source: PLoS One. 2020 Apr 24;15(4):e0229921. doi: 10.1371/journal.pone.0229921 (PMC7182175; doi:10.1371/journal.pone.0229921)

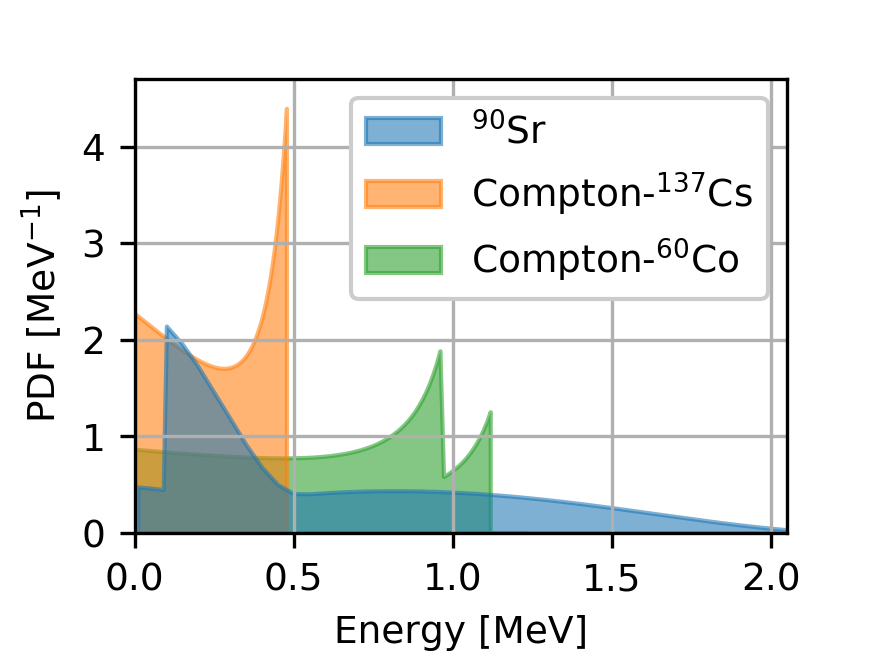

Supplement: S1 Fig — Comparison of Compton electron energy spectrum for the 137Cs and the 90Co to the β-spectrum of 90Sr. Compton electron spectra were calculated as as described in [45]. (TIF) [file pone.0229921.s002.tif]

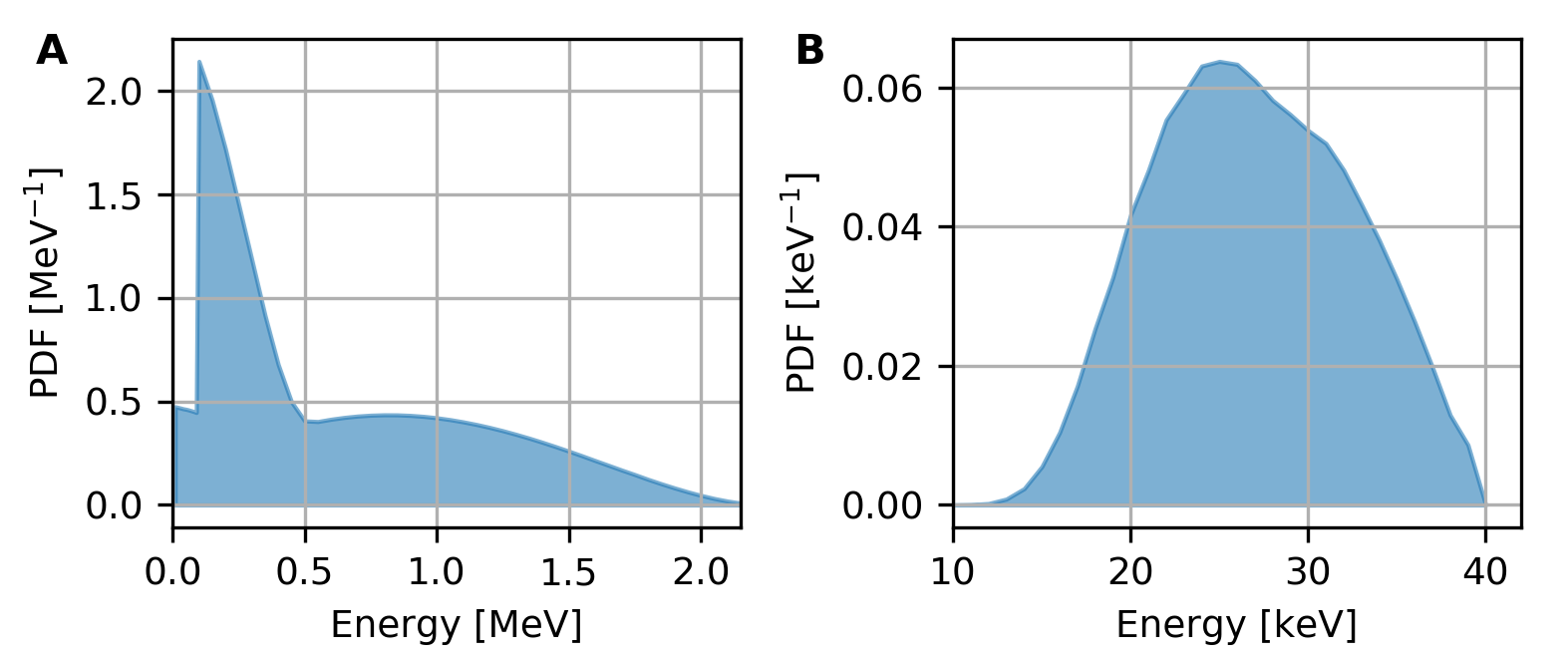

Supplement: S2 Fig — (A) Energy spectrum for the 90Sr source. (B) Energy spectrum for the 40 kVp X-ray source. (TIF) [file pone.0229921.s003.tif]

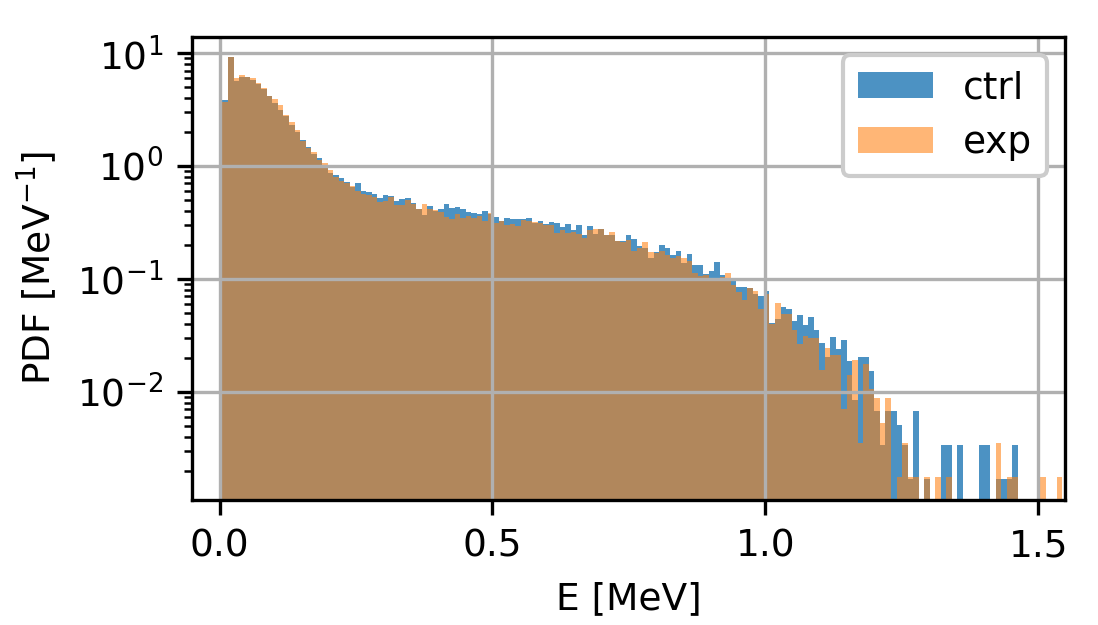

Supplement: S3 Fig — Comparison of the recorded spectrum for the S. officinalis and water suspension to its control (water only). (TIF) [file pone.0229921.s004.tif]

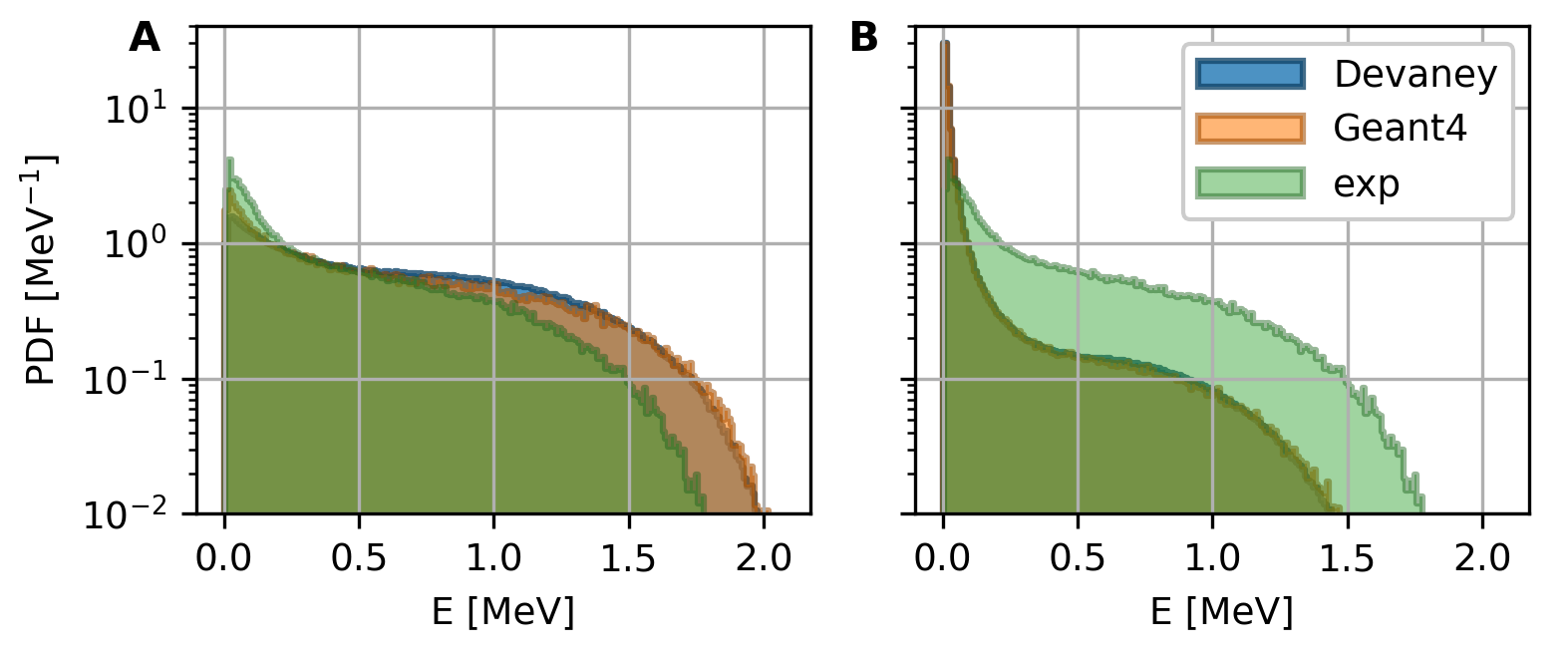

Supplement: S4 Fig — (A) Comparison between the simulated detector spectra, using the source spectrum from [29] (Devaney) or the Geant4 radioactive decay module (Geant4), for a detector that adds the contribution of the secondary particles to the primary and the experimentally recorded spectrum (exp). (B) Simulated spectra for a detector that registers each particle separately. The experimentally recorded spectrum is the same as in panel (A). (TIF) [file pone.0229921.s005.tif]

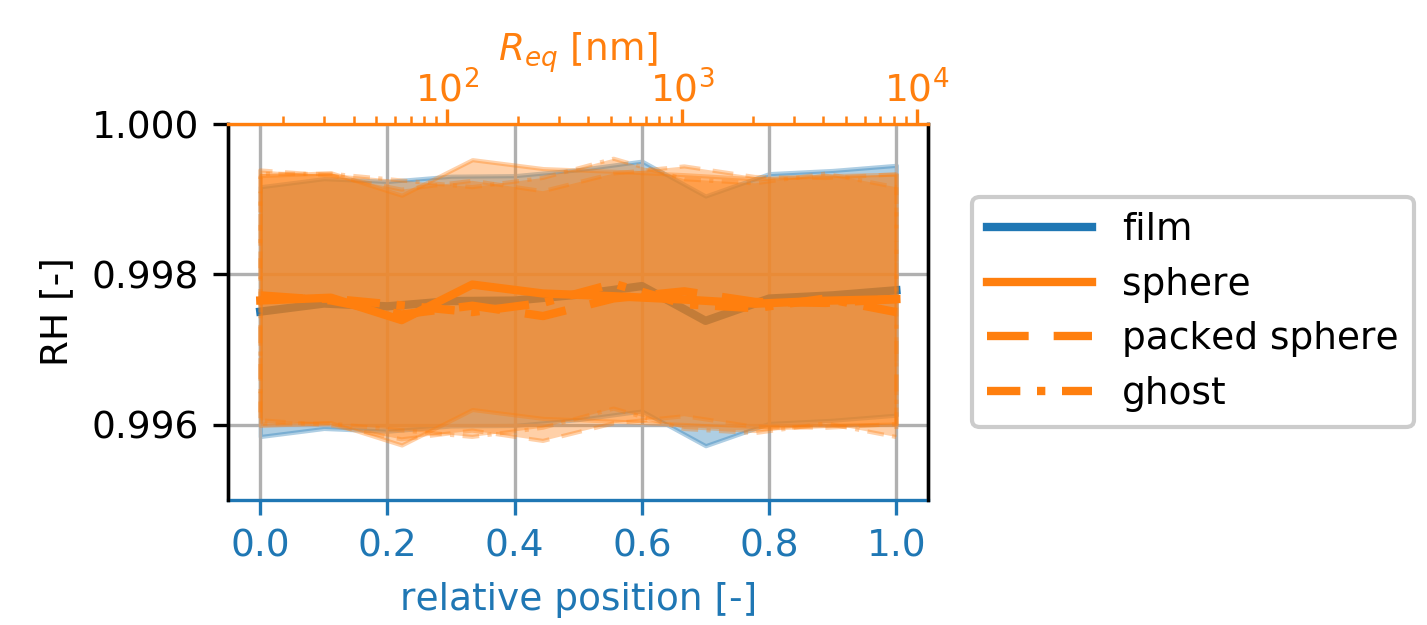

Supplement: S5 Fig — Relative radiant fluence for different spatial arrangements for the melanin-water composite and the X-ray source. The relative film position is marked on the bottom x-axis and the equivalent radius for the lattice configurations at the top logarithmic x-axis. (TIF) [file pone.0229921.s006.tif]
