# Supplementary material for: A biomimetic approach to shielding from ionizing radiation: The case of melanized fungi
Source: PLoS One. 2020 Apr 24;15(4):e0229921. doi: 10.1371/journal.pone.0229921 (PMC7182175; doi:10.1371/journal.pone.0229921)
Supplement: S1 Table — Elemental composition used in the numerical simulations for the synthetic and the S. officinalis melanins, and the cellulose. (PDF) [file pone.0229921.s007.pdf]

Table S1. Elemental composition of shielding materials

**Material**	**Element weight fraction** (wt.%)									
	C	H	N	O	Mg	Ca	K	Na	Cl	S
Mel. synthetic	51.40	2.76	6.66	34.24	-	-	4.92	-	-	-
Mel. *S. officinalis*	50.17	3.12	6.69	29.75	2.15	1.49	0.13	3.58	2.92	-
Cellulose	42.86	5.96	-	49.26	-	-	-	0.80	-	1.12
