# Supplementary material for: A biomimetic approach to shielding from ionizing radiation: The case of melanized fungi
Source: PLoS One. 2020 Apr 24;15(4):e0229921. doi: 10.1371/journal.pone.0229921 (PMC7182175; doi:10.1371/journal.pone.0229921)
Supplement: S2 Table — Summary of the samples with the corresponding controls and experimental campaigns. (PDF) [file pone.0229921.s008.pdf]

Table S2. Sample-control correspondence

Sample	Control	Campaign
Ammonia Mel#1	Ammonia #1	1
Ammonia Mel#2	Ammonia #1	1
Water Mel#1	Water #1	2,3
Water Mel#2	Water #2	1
Water CNC	Water #1	2,3
