# Supplementary material for: A biomimetic approach to shielding from ionizing radiation: The case of melanized fungi
Source: PLoS One. 2020 Apr 24;15(4):e0229921. doi: 10.1371/journal.pone.0229921 (PMC7182175; doi:10.1371/journal.pone.0229921)
Supplement: Attachment — Submitted filename: response_to_reviewers.pdf [file pone.0229921.s009.pdf]

Reviewer #1: The authors raise the possibility of using melanin as a shielding material against space radiation. The argument stems from published research suggesting that the extremely thin layer of melanin in a cell "shields" it from incoming ionizing radiation. To test this, they conduct shielding experiments with melanin and cellulose. They used low energy polyenergetic x-rays and polyenergetic beta particles from Sr-90 to conduct the experiments. The use of polyenergetic beams is not the best choice when conducting shielding studies. Monoenergetic beams are preferred. Furthermore, the type and energy of these radiations is nothing like those encountered in space. High-Z and high-energy particulate (HZE) radiations are encountered in space. These are extremely energetic and can penetrate even thick layers of titanium. Therefore, the thought that a thin layer of melanin has any useful physical shielding characteristics is a stretch. A quick study of stopping powers and attenuation coefficients would have answered this question without conducting experiments and modeling studies. With that in mind, it would be more helpful to focus on the biological mechanisms that may afford protection when melanin content is high.

Response: The manuscript discuss reported mechanisms to mitigate the effects of radiation damage, by investigating how melanized fungi manage to survive in high-radiation environments. Indeed, we fully agree with the reviewer that a thin film of melanin would be inadequate to protect against HZE. We make this also clear in the "Discussion" section of the manuscript, where we doubt that melanin is able to provide improved shielding even from the significantly less energetic β-radiation that we have employed in our experiments:

> l.368-370: "With respect to interaction of melanin with β-radiation, our experiments indicate that melanin does not exhibit improved shielding; the recorded RD for melanin was comparable to cellulose, a substance with similar chemical composition to melanin."

We want to point out that the manuscript *does* investigate one of the speculated biological mechanisms for the experimentally reported and published protective property of melanin; the physical interaction with the secondary β-particles. Fully consistent with published literature on the topic, the word "shielding" is used throughout the manuscript to indicate physical interaction of melanin with ionizing radiation, in contrast to biochemical, even if the melanin lies inside a living organism (a terminology that was not introduced by the authors). To make this point more clear we have added the following sentence in the Introduction:

> l.36-37 : "The word *shielding* is used here to indicate the physical interaction between radiation and melanin, even if the latter resides inside the cell."

Until today, it is unclear if this shielding plays a significant role, mainly because the vast majority of the previous experiments used model organism (animal or fungi cultures), where biochemical effects may mask physical and vice versa; by clearly separating the two, we hope that our experiment sheds some light on the matter.

Moreover, the shielding properties of melanin have caught the attention of the space community, which puts money and resources to pursue such potential; for example with the $200.000 project "Protecting Astronauts from Space Radiation-Induced Carcinogenesis and Central Nervous System Damage with Melanin-Containing Food and Materials" from the Canadian Space Agency [1] or the collaboration of John Hopkins University and NASA to send melanin samples to ISS [2,3].

For the aforementioned reason, the selected sources for our experiments do not intend to simulate the space radiation environment but to reproduce energy spectra from previous experiments; ^{90}Sr source imitates the Compton electron spectrum of ^{60}Co and ^{137}Cs sources (detailed Compton spectra derivation can be found in S1 Text – "Selection of radioactive source"). The X-ray spectrum is similar to previous experiments that connected melanin ghosts to increased attenuation of X-rays, as described in the "Spatial arrangement" subsection.

Further, the manuscript also proposes ways to improve radiation shielding materials; while it is trivial to estimate the shielding characteristics from stopping powers and attenuation coefficients for a single material (as the reviewer correctly points out), the same calculation for a composite material is a much more challenging and complex task. Our results suggest that arranging two material into different configurations may substantially change the shielding characteristics. Therefore, improved shielding effectiveness can be achieved for reduced shield mass, which is of interest in space flight. As we state in the manuscript, the superior shielding of composite materials has already been identified by the community, but to our knowledge, rationalization of the composite configurations or the use of micro- or nano-particle have not been discussed before in the literature.

Lastly, shielding experiments with monoenergetic beams have the value to characterize in detail shielding materials. Polyenergetic beams are able to resolve the magnitude of the attenuation of a given material and useful as a mean to assess if a monoenergetic experimental campaign is justified. As explained in the paper, we did not detect any significant effect of melanin in our polyenergetic experiments that support further investigation and we are confident that monoenergetic experiments will lead to the same conclusion.

References

[1] "Grants awarded under the FAST 2017 Announcement of Opportunity," 14 March 2019. [Online]. Available: https://www.asc-csa.gc.ca/eng/funding-programs/programs/fast/grants-awarded-fast-ao-2017.asp. [Accessed 30 January 2020].

[2] "Biological pigment that acts as nature's sunscreen set for space journey," Hub - Johns Hopkins University, [Online]. Available: https://hub.jhu.edu/2019/11/01/melanin-space-study/. [Accessed 13 February 2020].

[3] "NASA Tests Melanin-Based Radiation Blocker for Astronauts," Machine Design, [Online]. Available: https://www.machinedesign.com/materials/article/21838319/nasa-tests-melaninbased-radiation-blocker-for-astronauts. [Accessed 5 February 2020].

Reviewer #2: The work presented in the manuscript is very interesting and technically sound. Both the hypothesis about melanin shielding by beta radiation and spatial arrangement have been well presented by the experiments combined with simulation. Experimental results are backed by good statistically approaches.

The work can be published in the journal after the following comment has been addressed.
1. In the figure 3b, the experimental and simulation studies do not agree well in the low energy regime. Authors attribute it to 'spectrum of the 90Sr and neglected detector dynamics'. Authors need to explain why it affects only certain portion of spectrum. Explanation at present is weak.

Response: We have updated the manuscript in order to better explain the observed discrepancy between the ^{90}Sr spectra recorded during the experiments and simulation. For calibration, we had acquired spectra of the ^{90}Sr source without the melanin samples at the beginning of each experimental campaign. We simulated the same conditions in Geant4, namely the source and the detector (without the melanin sample) and we observed the same discrepancy between experiment and simulation; the experimental spectrum has more content in the low energy region. These result are shown in Figure 1A, with the experiment labeled as "exp" and the simulations are "Geant4". Therefore, we feel safe to conclude that this effect is not connect to the presence of melanin, the solvent or the container.

The most likely reason for the observed effect is the simplified model we used for the detector. We remind to the reviewer that for the simulations we have used the spectrum taken from [1] instead of fitting the recorded source spectrum to the observed one from the experiments, in an attempt to avoid any systematic errors. Further, we have modelled the detector using the following assumption: the detector registers the total deposited energy of the primary particle, that is the sum of the deposited energy from the primary and all the secondary particles. This assumptions inaccurately captures the exact detector dynamics, which is determined by the electronics and the signal processing algorithms that are applied to the voltage pulses induced in the crystal by the incoming particles. During the processing step, pulses can be merged or rejected, as stated in the manual [2]. To further investigate if these neglected dynamics are able to produce the magnitude of the observed shift, we modelled the detector that is able to record any secondary particle as a separate event, independent from the primary that created it. As before, we used the spectrum from [1] and the radioactive decay module of Geant4. The latter simulates an immobile ^{90}Sr nuclei and tracks all the intermediate nuclear reactions and products, making it computationally more demanding. It is expected that this detector model will shift the content of the histogram towards lower energies. The comparison of the two detector models is shown in Figure 1. Firstly, both the spectrum from Devaney and the radioactive decay module of Geant4 produce similar results. More importantly, the neglected detector dynamics are able to produce the observed effect between simulation and experiment, with the real detector response most likely lying somewhere between these two extreme cases.

The way we have selected to model the detector seems to sacrifice some of the accuracy of the final spectrum, but it captures the overall trend. Moreover, the dose calculation is not affected by the detector modelling, since in the simulations the production and tracking of the secondary particles do not depend on the way they are binned in the histogram. We only expect a minor effect on the estimation of the dose variance, but we remark that we never use statistical comparisons that mix simulations and experiments. The implemented detector model is justified by the tradeoff between accuracy versus complexity and execution time.

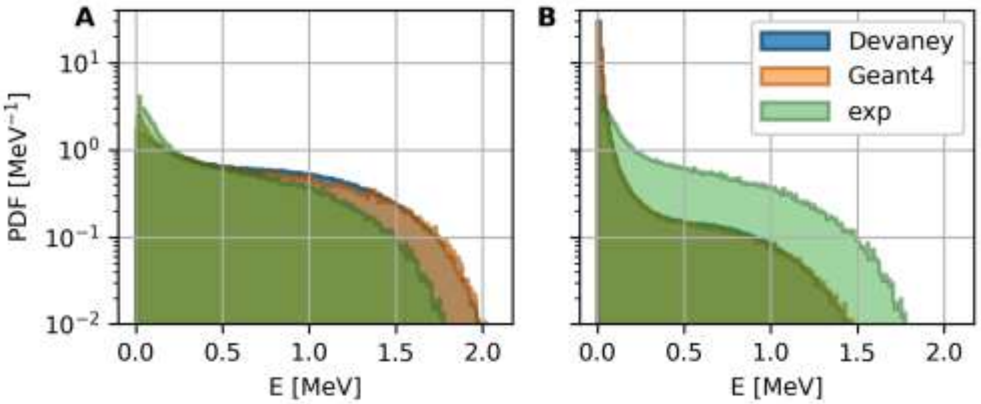

Figure 1: Simulated ^{90}Sr spectra for two detector models. (A) Comparison between the simulated detector spectra, using the source spectrum from [1] (Devaney) or the Geant4 radioactive decay module (Geant4), for a detector that adds the contribution of the secondary particles to the primary and the experimentally recorded spectrum (exp). (B) Simulated spectra for a detector that registers each particle separately. The experimentally recorded spectrum is the same as in panel (A).

The previous discussion and Figure 1 have been included to the supplementary materials. The text in manuscript has be altered to direct the reader to the added SI section. The code that produce the discussed simulations and Figure 1 has been added to the repository.

References

[1] J. J. Devaney, "Beta spectra of ^{90}Sr and ^{90}Y," Los Alamos National Lab, 1985.

[2] *CUBE527 Spectrometer series - User Manual,* Radeberg: GBS-Elektronik GmbH, 2018.

Reviewer #3: This is an interesting study. The manuscript is written well and I am happy to recommend acceptance of the study. I would like the authors to make a minor change, Lines 310 - 314 ("For the spatial arrangement hypothesis, we used melanin (synthetic, see S1 Table for elemental composition) and water as the high-Z and low-Z materials respectively. We fixed the geometric parameters of the composite shield at A = 3:37 mg cm2 and RV = 0:234. We simulated various configuration for hr from 0 to 1 and Req from 16nm to 8192nm with the 90Sr and the 40 kVp X-ray source") may be transferred to the methods section.

Response: We have moved the specific text to the methods section, as proposed by the reviewer.
